# Convergent morphological responses to loss of flight in rails (Aves: Rallidae)

**DOI:** 10.1002/ece3.6298

**Published:** 2020-06-01

**Authors:** Julien Gaspar, Gillian C. Gibb, Steve A. Trewick

**Affiliations:** ^1^ Wildlife & Ecology Group School of Agriculture and Environment Massey University Palmerston North New Zealand

**Keywords:** birds, evolution, flightlessness, island ecology, morphological convergence, *Rallidae*

## Abstract

The physiological demands of flight exert strong selection pressure on avian morphology and so it is to be expected that the evolutionary loss of flight capacity would involve profound changes in traits. Here, we investigate morphological consequences of flightlessness in a bird family where the condition has evolved repeatedly. The Rallidae include more than 130 recognized species of which over 30 are flightless. Morphological and molecular phylogenetic data were used here to compare species with and without the ability to fly in order to determine major phenotypic effects of the transition from flighted to flightless. We find statistical support for similar morphological response among unrelated flightless lineages, characterized by a shift in energy allocation from the forelimbs to the hindlimbs. Indeed, flightless birds exhibit smaller sterna and wings than flighted taxa in the same family along with wider pelves and more robust femora. Phylogenetic signal tests demonstrate that those differences are independent of phylogeny and instead demonstrate convergent morphological adaptation associated with a walking ecology. We found too that morphological variation was greater among flightless rails than flighted ones, suggesting that relaxation of physiological demands during the transition to flightlessness frees morphological traits to evolve in response to more varied ecological opportunities.

## Graphical abstract

This study investigated the consequences of the evolution of flightlessness in birds from the Rallidae family. The results show similar morphological responses within the flightless species with a shift in energy allocation from the forelimbs to the hindlimbs. By testing the phylogenetic signal, this research proves that the differences between flightless and flighted birds are not an effect of the phylogeny but reflect a morphological convergent adaptation to the loss of flight.

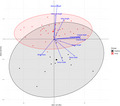

## 1 INTRODUCTION

Living neoaves include more than 10,000 extant species around the world in many different habitats (Brusatte, O'Connor, & Jarvis, 2015). An almost universal feature of this diversity is a reliance on aerial flight.

Studies of morphological evolution based on fossil evidence showed that birds developed laterally wide and robust oriented forelimbs along with a large extension of the sternum called a keel and powerful pectoral muscles in order to make flapping flight possible (Roots, 2006; Xu et al., 2014). Their bodies also became smaller and streamlined (Turner, Pol, Clarke, Erickson, & Norell, 2007), and their bones and muscles evolved to generate powered flight for a reduced weight (Roots, 2006). Flight is energetically demanding which appears to be one of the reasons for the relatively high metabolic rate in birds compared with reptiles and mammals (Maina, 2006; Møller, 2009). For instance, a bird expends around 75% more energy during one day than a terrestrial mammal of similar size (Maina, 2006).

### 1.1 Flightlessness in birds

Flight demands significantly impact the morphological and physiological characters in birds (Elliott et al., 2013). Indeed, this ability has a substantial energetic cost which leads to many constraints in terms of body size, weight, reproduction, shape etc. (McNab, 1994). Such constraints exert intense ecological trade‐offs (Alexander, 1998; Lighthill, 1975; Rayner, 1988; Ricklefs, 1973). Therefore, flightlessness can be positively selected in an environment where the flight does not provide a significant benefit. This can lead to conservation issues if the habitat changes swiftly and the flight is required again. For example, if predators are introduced, flightless birds could be unable to avoid them.

Transitions to flightlessness are considered rapid and irreversible (Kirchman, 2009; McNab, 1994; Slikas, Olson, & Fleischer, 2002) and have occurred independently in more than 20 avian families (Roff, 1994). A notable example is the ratites (including ostriches, kiwi, and emus), a polyphyletic group characterized by multiple independent loss of flight (Harshman et al., 2008; Phillips, Gibb, Crimp, & Penny, 2010).

Flightlessness has been observed in many island species and is interpreted as an effect of the insular conditions which often provides an habitat with few or no predators and limited competition for resources (McNab, 1994). Flightlessness evolves most frequently in island birds that belong to lineages for which flight is not essential for foraging, and are released from the need to escape predators (McNab, 1994; Olson, 1973). On islands with reduced raptor species richness and no mammalian predators, birds evolve smaller flight muscles, consistent with selection for flightlessness (Wright, Steadman, & Witt, 2016). The loss of flight removes many constraints in terms of weight and body size leading to significant morphological changes (Livezey, 2003). For instance, many flightless birds are larger than their flighted relatives (Roots, 2006). The most prominent examples are the ostrich which stands 2.5 m tall, and the recently extinct 2‐m high South Island giant moa (*Dinornis robustus*). Nevertheless, flightless species have a great size range. Some of them are small compared with their flying relatives like the 12.5 cm‐long Inaccessible Island rail (*Atlantisia rogersi*; Roots, 2006).

The rails or Rallidae are a family of birds that diversified during the Eocene around 40 million years ago (Garcia‐R, Gibb, & Trewick, 2014b) and includes around 130 species among which over 30 are (or were, for recently extinct species) flightless (Garcia‐R, Gibb, & Trewick, 2014a; Kirchman, 2012; Steadman, 1995). Despite the fact that many rails have a terrestrial lifestyle (Taylor, 1998), some lineages have a tendency to colonize oceanic islands (Olson, 1973; Ripley, Lansdowne, & Olson, 1977) resulting in a wide representation around the world. Fossil records show that extensive late Quaternary extinction within this group resulted from human colonization of islands (Steadman, 2006). The majority of the flightless birds within this family are endemic to single islands, which implies that in most of the cases, their ancestors had to be flighted to reach this habitat as most of the islands were never connected to continental landmasses (Trewick, 1996, 1997a, 1997b).

Qualitative and morphometric analyses of flighted and flightless rails suggest that transition to flightlessness in rails often involves some common traits, but the phylogenetic hypothesis used to examine transitions to flightlessness relied on many of the same morphological characters (Livezey, 2003). We now know that the morphological phenogram (Livezey, 2003) poorly represents many evolutionary relationships within the family possibly reflecting morphological convergence associated with flightlessness (Garcia‐R et al., 2014a).

Phylogenetic analyses based on five genes (three mitochondrial and two nuclear) show that rails are separated in eight clades: *Fulica, Aramides*, *Porphyrio, Rallina, Porzana, Laterallus, Gallicrex,* and *Rallus* (Garcia‐R et al., 2014a). Four of these clades contain flightless species (*Fulica*, *Gallicrex, Porphyrio,* and *Rallus*), and this is particularly pronounced in the *Rallus* clade where a majority of sampled birds are flightless. Here, we use a modern and independent molecular phylogenetic hypothesis for the rails to investigate morphological evolution of flightlessness in the rail family, among which repeated loss of flight could yield convergent morphological evolution.

## 2 METHODS

### 2.1 Datasets

#### 2.1.1 Morphological data

We assembled a matrix that includes 10 morphological traits for 90 species including extant taxa and those that went extinct after they were first described (Livezey, 2003), (Appendix 1: Table A1). The selected traits are among the most commonly used in the literature concerning morphological differences between flighted and flightless birds (Cubo & Arthur, 2001; Lambertz & Perry, 2015; Livezey, 1992; Roots, 2006; Trewick, 1997b). These data were supplemented by the standard body lengths of rails reported in the Handbook of the Birds of the World Alive Online (del Hoyo, Elliott, Sargatal, Christie, & de Juana, 2015). Mean metric values were used when data from different individuals, or a range of values, were available. The amount of missing values in the full dataset is close to 32%.

The taxonomy used in this study follows the “Clements Checklist 2018” (Clements et al., 2018), so some of the names presented in Livezey (2003) have been modified accordingly.

Each species was characterized as a flighted or flightless species according to Taylor (1998) or Garcia‐R et al. (2014a). Other information including the distribution and habitat was added to the dataset based on Garcia‐R et al. (2014a). A subset of the data was created including only those species for which molecular phylogenetic information was available (Appendix 1: Table A2). This subset included 52 species and 11 morphological traits: body length, wing length (chord of the flattened wing), body mass, cranial length, cranial depth, cranial width, sternum length (the length of the extension of the sternum called keel or carina), sternum depth (perpendicular depth of the keel), pelvis width (interacetabular width), femur length, and femur width (the width of femoral head or caput) and contains only 11% missing values.

We treated the purple swamphens (genus *Porphyrio*): *P. bellus, P. melanopterus, P. melanotus, P. melanotus ellioti, P. poliocephalus, P. porphyrio, P. pulverulentus, P. samoensis,* considered by Livezey (2003) as different species, as a single taxon: *Porphyrio porphyrio* (Garcia‐R & Trewick, 2014). Mean metric values (when data were available) were used to determine *P. porhyrio* morphological data.

#### 2.1.2 Molecular data

Molecular data are available for 88 rail species and seven outgroup species. Five genetic markers were used including 3 mitochondrial genes (COI, cyt‐b, 16S) and 2 nuclear genes (FGB, RAG‐1) from Garcia‐R et al. (2014a) (NCBI accession numbers available in Appendix 1: Table A3). The number of available sequences per gene varies between 64 (FGB) and 85 (cyt‐b).

### 2.2 Analysis.

#### 2.2.1 Phylogenetics

**FIGURE A1:**
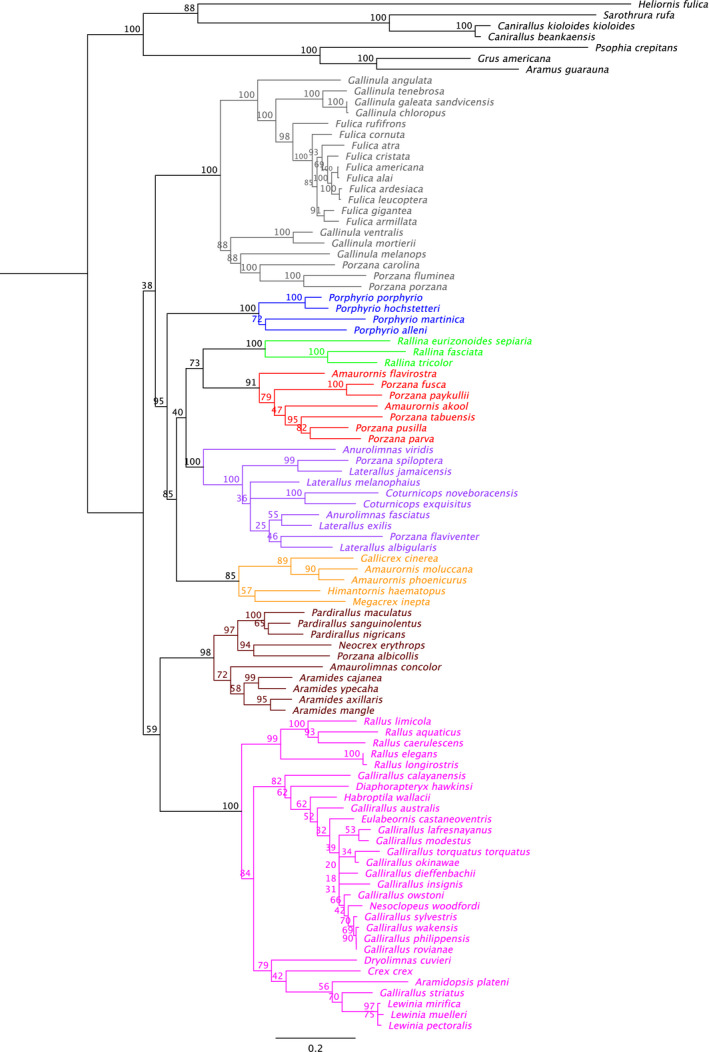
Maximum likelihood phylogeny based on a 5‐gene (COI, cyt‐b, 16S, FGB, RAG‐1) concatenated analysis, bootstrap supports are indicated in each branch. Colours are indicating the taxon according to Garcia‐R et al. (2014a), Dark grey: Fulica; Blue: Porphyrio; Green: Rallina; Red: Porzana; Purple: Laterallus; Orange: Gallicrex; Brown: Aramides; Pink: Rallus

Phylogenetic inference was tailored to the different phylogenetic signal tests we undertook. For each of the five genes, the sequences were independently aligned (Geneious Alignment, free gaps, 65% similarity) using the software Geneious 11.1.4 (https://www.geneious.com) then concatenated into a single alignment (see supplementary data). The alignment was processed using PartitionFinder2 (Lanfear, Frandsen, Wright, Senfeld, & Calcott, 2017) via the Cipres portal (Miller, Pfeiffer, & Schwartz, 2010) to select the best partitioning scheme and associated models of molecular evolution as follows: 16S: GTR + I+G; COI first codon positions: GTR + I+G; COI second codon positions: TVM + I+G; COI third codon positions: TIM + G; cyt‐b first codon positions: TVM + I+G; cyt‐b second and third codon positions: GTR + I+G; FGB7: TVM + G; RAG1 first codon positions: GTR; RAG1 second codon positions: HKY + I; RAG1 third codon positions: SYM + G. Maximum likelihood (ML) analyses were implemented in RAxML v8.2.10 (Stamatakis, 2014) via the CIPRES Science Gateway (Miller et al., 2010) with bootstrapping automatically stopped employing the majority rule criterion. The consensus tree was then visualized in Geneious (Appendix 1: Figure A1). All available rail data (88 rails plus 7 outgroup species) were used to create the phylogenetic tree which was then pruned down to the subset of 52 rail species for which morphological data were available. This 52 taxa tree was used for all downstream analyses. Discrete traits (e.g., habitat and the ability to fly) were mapped to that tree using R package phytools (Revell, 2012). The same tree was used in association with the results of the PCA on morphological data to generate a graph of phylomorphospace depicting the projection of a phylogenetic tree within the two first dimensions of a principal component analysis.

#### 2.2.2 Statistics

**FIGURE A2:**
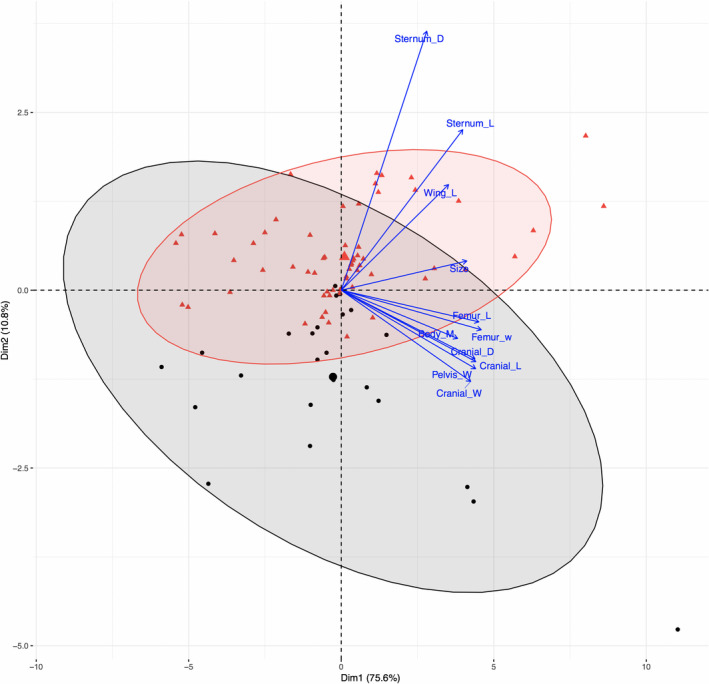
Principal component analysis (PCA) plot showing the two first dimension of the multivariate variation among 90 species of rails in terms of morphological traits. Vectors indicate the direction and strength of each trait contribution to the overall distribution. Black dots represent flightless species and red triangles are flighted species

Statistical analysis was performed in R (R Core Team, 2014; the script is available in supplementary data) using the following packages:, FactorMineR (Lê & Husson, 2008), car (Fox & Weisberg, 2018), phytools (Revell, 2012), ggplot2 (Wickham, 2011), and phylosignal (Keck, Rimet, Bouchez, & Franc, 2016). A first principal component analysis (PCA) on 90 species (65 flying and 25 flightless) was performed to observe the variation within the rail group and to determine the importance of the different traits and their correlation (Appendix 1: Figure A2). This analysis revealed a high level of correlation between all the morphological traits (Appendix 1: Figure A2). After detecting a significant correlation between the trait “Body length” and the first dimension of the PCA (that covers 75.6% of the variance) using a linear model (*F*(1, 67) = 244.7, *p* < .000, *R* ^2^ = .78), a correction was applied to dataset by dividing each trait by the body length of the relevant species. This standardization of the dataset allowed us to analyze the differences in the overall body shape between flighted and flightless rails rather than to compare the actual size of each body part. Thus, the corrected dataset represents a ratio of each trait compared the body length of each species. The body mass was log‐transformed as the distribution of that trait was not normally distributed.

A subset of the data for the 52 species with phylogenetic information was generated and contained a lower frequency of missing values (11% compared to 32% in the 90 species dataset). For each trait, a phylogenetic hypothesis was obtained by pruning the full phylogeny as appropriate to represent only the species for which the trait values were available for that trait. The phylogenetic signal was quantified using Blomberg's *K* statistic (Blomberg, Garland, & Ives, 2003), which estimates the phylogenetic signal (branch length) using the morphological trait variance relative to an expectation under a Brownian motion null model of evolution. A K values less than one imply that relatives resemble each other less than would be expected under Brownian motion evolution across the candidate tree.

PCA on the dataset of 52 species dataset was performed after replacing the remaining missing values within the matrix by the average value of the available data for each trait. Coordinates from the three first dimensions were used to evaluate variance differences between the groups. We used a Shapiro–Wilk test to determine the normality of each distribution and then performed *F* tests if the distribution was normal or Levene's test if it was not.

Bivariate correlation plots were then used to visualize patterns associated with flight ability including all the species for which the “Body length” value was available (75 species) were used in that analysis.

Major differences between flightless and volant species were observed in the correlations involving traits associated with flight and traits associated with walking. To investigate this phenomenon, a 52 species dataset of ratios was created by dividing the trait values from the upper part of the body (sternum depth and wing length) by the trait values from the lower part of the body (pelvis width and femur length) and body mass. Body length divided by body mass and sternum depth divided by sternum length were also investigated. t Tests were used to compare flighted and flightless birds on different trait ratios.

Binary logistic regression was performed on the data for 52 species to evaluate the influence of each trait on the character “Flying.” In order to minimize loss of information resulting from missing values, this analysis was performed independently for each of the 10 traits.

### 2.3 Data deposition

Data available from the Dryad Digital Repository: https://doi.org/10.5061/dryad.dz08kprsz.

## 3 RESULTS

### 3.1 Trait correlations

**FIGURE 1:**
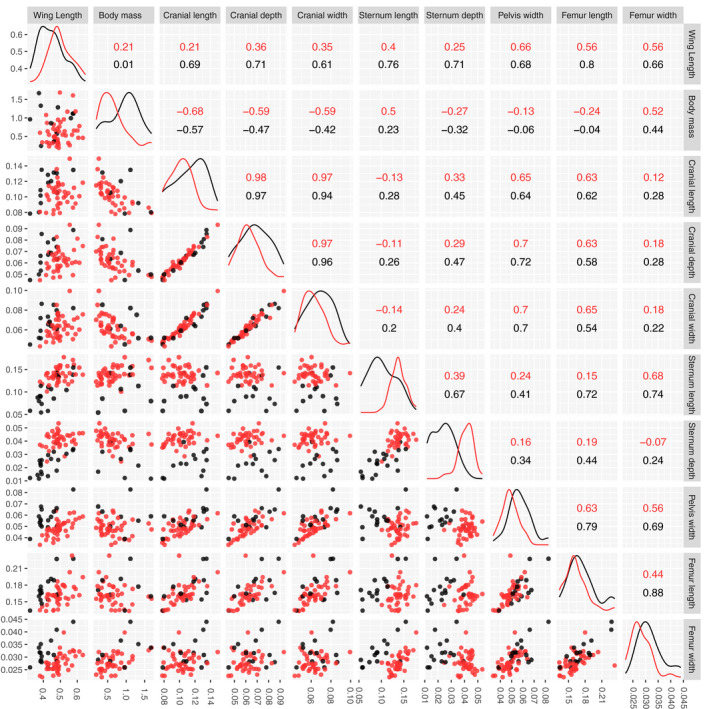
Scatter plot matrix of 10 traits (corrected by body length) from 75 rail species (those for which the body length is available), 49 flying and 26 flightless. The upper part of the diagonal shows the coefficient of determination (*r* ^2^) for flightless (black) and flying (red) species. Two traits are considered highly correlated when the coefficient is close to one. The lower part of the diagonal shows the scatter plots for each pair of traits and the diagonal shows the distribution of the values for each group

A scatter plot matrix of ten traits was used to visualize patterns associated with flight (Figure 1), although the number of species for each correlation was not constant due to some missing values. For some traits, differences between the flying group (red) and the flightless group (black) were readily apparent observed from the density plots (Figure 1 on the diagonal); the most obvious being body mass and sternum depth. Scatterplots of the three cranial measurements showed, as expected, that they were correlated with one another despite no difference between flighted and flightless taxa. Among other traits, scatterplot clustering and group differences were mostly observed where sternum depth and, to a lesser extent, sternum length were included. Wing length when compared with leg traits (pelvis width, femur length, and width) also exhibited differences between flighted and flightless groups. Broadly speaking, group differences were observed in plots of traits associated with flight (wing length and sternum depth) and traits associated with walking (pelvis width, femur length, and femur width). Finally, we note that the evolution of the sternum depth relative to the sternum length presents group clustering along a similar slope for both groups.

### 3.2 Principal component analysis

**FIGURE 2:**
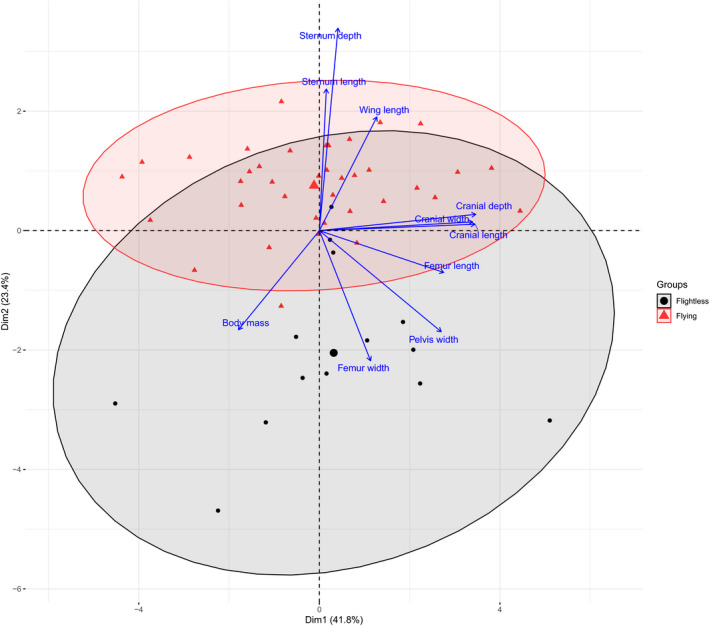
Principal component analysis (PCA) plot showing the two first dimension of the multivariate variation among 52 species of rails in terms of morphological traits. Vectors indicate the direction and strength of each trait contribution to the overall distribution. Black dots represent flightless species and red triangles are flighted species. 95% confidence ellipses are displayed (red for flying rails, black for flightless rails), a larger ellipse is associated with a high group variance

A principal component analysis (PCA) was performed on the 52 species dataset including 14 flightless and 38 flighted rails using the 10 traits (Figure 2). The two first principal components (Figure 2) explained 41.8% and 23.4% of the variance, respectively (Table 1). PC1 was mostly influenced by cranial length, depth, and width contributing 21%, 21%, and 20% of the variance respectively, and PC2 by sternum depth (36%), sternum length (17%), and femur width (16%; see Table 2). Flighted and flightless species clustered separately with flighted taxa mostly in the upper part of the plot and most flightless species in the lower part. The distinction between these groups was therefore mainly explained by the second principal component (the vertical dimension on the plot).

**TABLE 1:** Variance explained by each of the first three dimensions in the principal component analysis

	Dimension 1	Dimension 2	Dimension 3
Variance	4.177	2.338	2.217
Percentage of variances explained	41.767	23.381	22.166
Cumulative percentage	41.767	65.148	87.314
Variance test			
Statistic	1.26	7.16	1.084
Significance	0.56	0.010*	0.803

Note

The variance test implies a null hypothesis that the two groups (flying and flightless) have the same variance. An *F* test is used when the distribution is normal (Dimensions 1 and 3) and Levene's test when the distribution is not normal (Dimension 2).

**TABLE 2:** Variance explained by each variable in the principal component analysis

Trait	Dimension 1			Dimension 2			Dimension 3		
	Coord.	Contrib.	cos2	Coord.	Contrib.	cos2	Coord.	Contrib.	cos2
Wing length	0.352	2.974	0.124	0.5	10.673	0.25	0.631	17.952	0.398
Body mass	−0.488	5.7	0.238	−0.466	9.27	0.217	0.642	18.616	0.413
Cranial length	0.936	20.989	0.877	0.031	0.04	0.001	−0.282	3.598	0.08
Cranial depth	0.939	21.104	0.881	0.078	0.258	0.006	−0.253	2.89	0.064
Cranial width	0.922	20.357	0.85	0.036	0.056	0.001	−0.212	2.036	0.045
Sternum length	0.052	0.064	0.003	0.623	16.607	0.388	0.684	21.13	0.468
Sternum depth	0.124	0.369	0.015	0.913	35.637	0.833	0.258	3.002	0.067
Pelvis width	0.728	12.699	0.53	−0.464	9.199	0.215	0.341	5.249	0.116
Femur length	0.751	13.516	0.565	−0.209	1.866	0.044	0.362	5.916	0.131
Femur width	0.305	2.227	0.093	−0.619	16.396	0.383	0.659	19.611	0.435

Note

Coord. = Coordinate indicates (from 0 to 1) the correlation between the variable and the principal component; Contrib. = Contribution is a percentage of how much each trait explains the variance and cos2 (= Coord. * Coord.) is used to estimate the quality of the representation.

Generally, the ability to fly was positively correlated with the sternum depth and length and with the wing length. The flightless rails generally had wider femora and pelves and a heavier body. Cranial traits did not seem to be discriminant variables. Although the flightless group had fewer species, its variance and the 95% confidence ellipse appeared larger than the flighted group. To test that, a variance test was run on each of the three first dimensions. Variances in flighted and flightless group were not significantly different in dimension 1 and 3, but in dimension 2, the variance of the flightless group was significantly higher than in the flying one (Table 1).

### 3.3 Logistic regression

Logistic regressions revealed that five of the ten analyzed traits had a significant effect on the “flying” character: wing length, sternum length, sternum depth, pelvis width, and femur width (Table 3). The regression coefficients were positive for the wing length, the sternum length, and the sternum depth but negative for pelvis width and femur width. This means that the possibility of being flighted increases when the wing length and the sternum size increase but decreases when the pelvis and femur width are large.

**TABLE 3:** Logistic regression performed on a subset of the 52 species dataset showing the relationship between 10 morphological traits and the ability to fly

Trait	Number of species		Logistic regression			Phylo. signal
	Flying	Flightless	Coefficient	Statistic	Significance	Blomberg's *K*
Wing length	38	14	27.696	3.194	0.001***	0.322
Body Mass (log)	38	11	−1.369	−1.581	0.114	0.493
Cranial length	33	9	−21.834	−0.870	0.384	0.348
Cranial depth	33	9	−16.101	−0.392	0.695	0.384
Cranial width	33	9	−33.426	−0.958	0.338	0.388
Sternum length	35	10	126.575	2.958	0.003**	0.279
Sternum depth	35	10	530.465	2.383	0.017*	0.290
Pelvis width	35	10	−179.97	−2.469	0.013*	0.310
Femur length	35	10	−17.808	−1.123	0.261	0.279
Femur width	35	10	−255.919	−2.589	0.009**	0.276

Note

Asterisks show significance of *p*‐values; * *p* < .05, ** *p* < .01, *** *p* < .001. A *p*‐value under .05 for the normality test (Shapiro–Wilk) indicates the null hypothesis that the sample is normally distributed is rejected. Blomberg's *K* measures the phylogenetic signal, if it is <1 the variable is phylogenetically independent.

### 3.4 Ratio comparison

**FIGURE 3:**
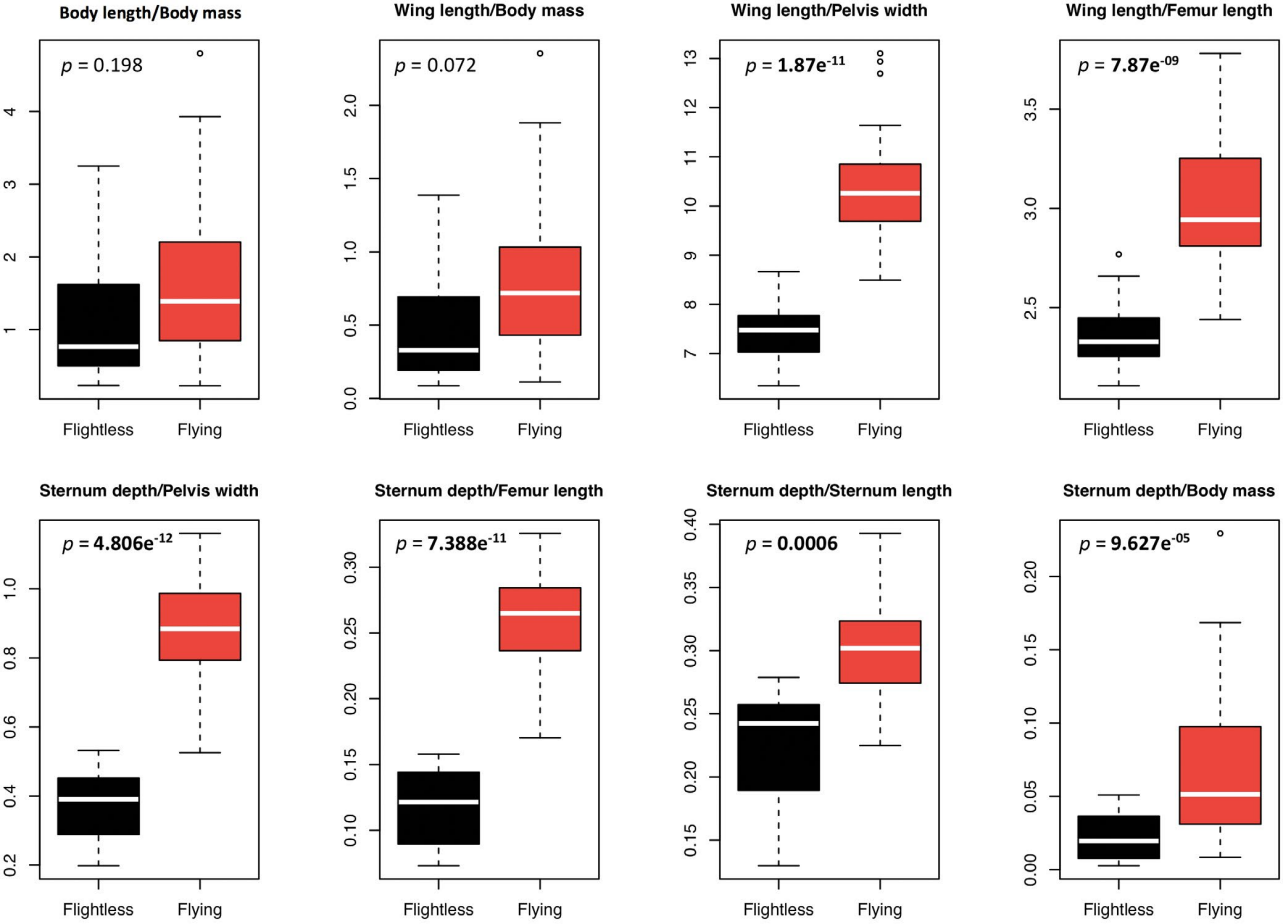
Comparison of ratio values for flighted (red) and flightless (black) groups. Ratios were created by dividing the trait values from the upper part of the body (sternum depth and wing length) by the trait values from the lower part of the body (pelvis width and femur length) and body mass. Body length divided by body mass, wing length divided by body mass, sternum depth divided by sternum length and sternum depth divided by body mass are shown as well. *p*‐values below .05 indicate a significant group difference (*T*‐test)

The flighted group showed significantly higher ratio values in all the comparisons except two, body length divided by body mass and wing length divided by body mass (Figure 3). This was expected as traits associated with flight should be higher in flighted rails. We note that the ratio between the depth and the length of the sternum showed significant group difference. This suggests that a single bone may give an indication regarding the flight capacity of a bird, although the ratio values between flightless and volant groups overlap. The flightless group always had a lower ratio value when a trait associated with walking was involved.

### 3.5 Phylogenetic tree

A maximum likelihood phylogeny was generated using 5 genes and 95 birds species (88 rails and 7 birds from other families as an outgroup; Appendix 1: Figure A1). Maximum likelihood bootstrap support was largely consistent with the phylogeny of Garcia‐R et al. (2014a).

**FIGURE 4:**
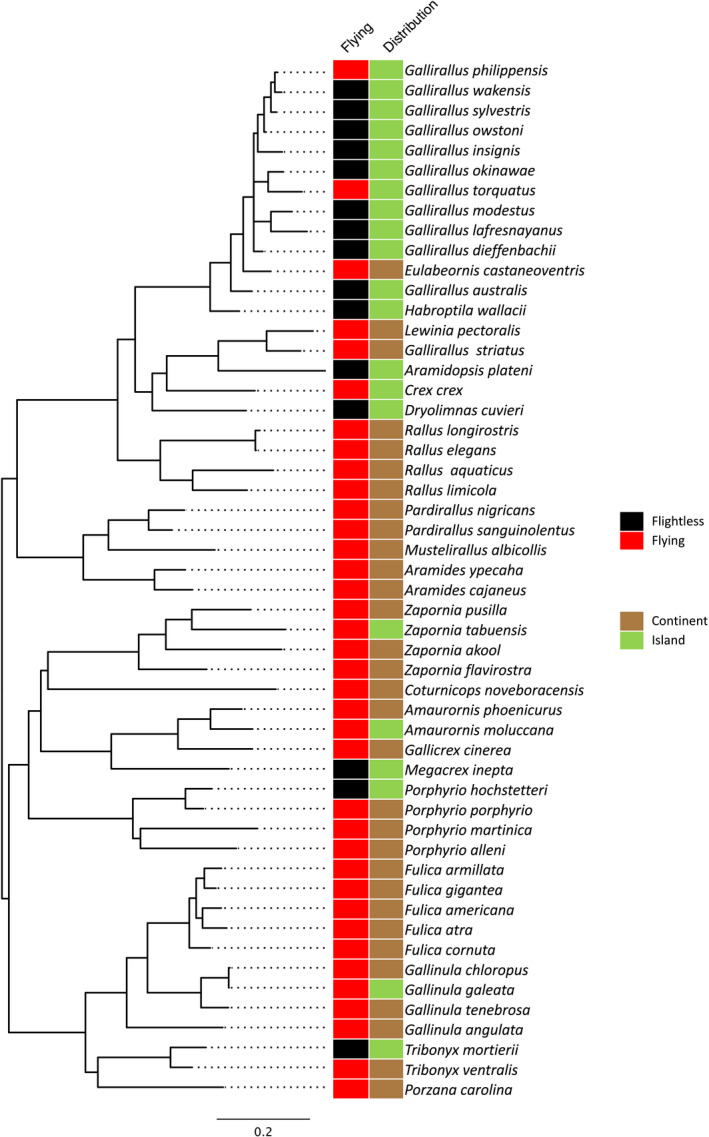
Flying ability and distribution (island or continent) mapped on the 52 species Maximum likelihood phylogenetic tree of rails

A subset of the phylogenetic tree was obtained comprising only the species for which we had morphological data (Figure 4). Flying ability and the geographic distribution of each species were also mapped on this tree. The majority of available species (38) in the analysis were classified as flying and of the flightless ones (14 species) many were in the *Rallus* group although *Fulica, Gallicrex,* and *Porphyrio* each have one flightless species.

When the ability to fly was compared with the habitat of the different species, a clear relationship is observed between the flightless trait and island habitat (Figure 4). Indeed, all 14 flightless species represented in the phylogenetic tree live on islands, although islands differ in terms of the habitat they provide. For instance, *Gallirallus modestus* is endemic to the small Chatham Islands, while other species including *Porphyrio hochstetteri* and *Dryolimna cuiveri* inhabit the larger continental islands, New Zealand, and Madagascar, respectively.

**FIGURE 5:**
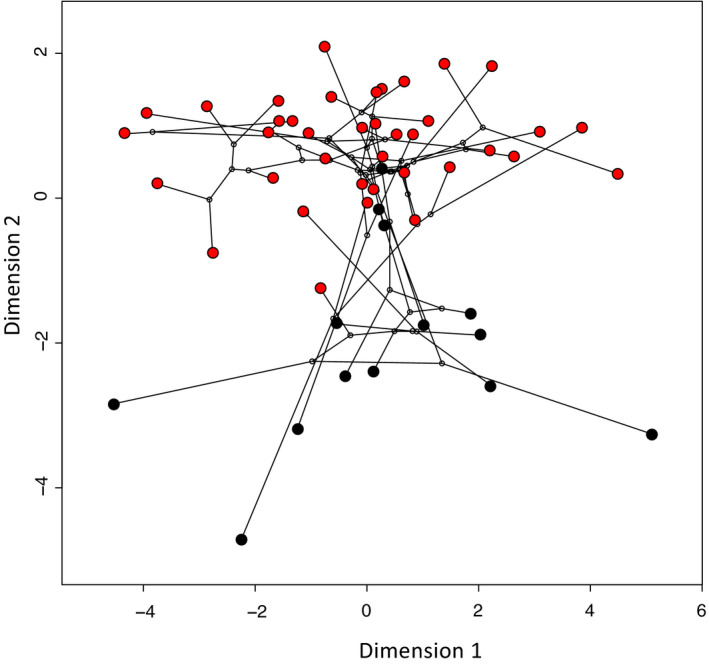
Phylomorphospace. Projection of the 52 species Maximum likelihood phylogenetic tree of rails within the two first principal component of a PCA performed on 10 morphological traits. Black dots indicate flightless species and red dots indicate flying species. Black lines indicate phylogenetic relationships between species

The phylogenetic tree of 52 species was used to quantify the phylogenetic signal of each morphological trait using Blomberg's K (Table 3). All ten traits tested showed K value lower than 1 suggesting phylogenetic relatives resemble each other less than expected under Brownian motion evolution along the candidate tree (Blomberg et al., 2003). These K values imply the evolution of the morphological traits is uncorrelated with phylogeny. Data from the principal component analysis and phylogenetical analysis for 52 species were then combined to produce a phylomorphospace graph (Figure 5), which suggests that the clustering observed in the morphospace (PCA result, Figure 2) was not correlated with the phylogenetic tree as multiple branches extend between the flighted and the flightless group.

## 4 DISCUSSION

### 4.1 Morphological differences between flighted and flightless rails

Trait correlation analysis showed multiple trend differences between flighted and flightless groups. Such differences were also observed in principal component analysis with clear group clustering, confirming the existence of a strong link between the flight ability and phenotypic evolution in rails (Livezey, 2003). This phenomenon has now been observed in several bird families (Baker, Haddrath, McPherson, & Cloutier, 2014; Diamond, 1991; Gussekloo & Cubo, 2013).

Results from logistic regression suggest that the transition from flighted to flightless involves a reduction of the sternum depth and length together with a shortening of the wing length. Flightless rails also exhibit wider pelves and femora which is consistent with the informal description of flightless birds as species with bigger feet, legs, and leg muscles to support a heavier body (Roots, 2006). The pattern revealed by logistic regression was also from PCA graph (Figure 2) where the distinction between groups is mostly present on the second dimension (covering 23.4% of the variance). The variables mostly influencing the variance within that component are, by order of importance, sternum depth, sternum length, femur width, wing length, body mass, and pelvis width. This is consistent with the inference that, in rails, the transition to flightlessness usually involves an increase in body size, body mass, pelvis, and cranium size as well as a reduction in lengths of wings and sternum size (Livezey, 2003). The fact that body mass seems to be associated with the ability to fly in the PCA but is not statistically significant in the logistic regression is probably due to the high variance of that trait. Some flightless rails exhibit relatively high body mass (McNab, 1994; Roots, 2006) but other are much smaller. In New Zealand, for example, the flightless takahe *Porphyrio hochsteterri* (2.7 kg) is about 450 times heavier than Gallirallus modestus (60 g) rails living in the New Zealand.

We found no correlation between cranial traits and the ability to fly using logistic regression as well as PCA confirming our expectation that these characters are not directly linked to flight or walking efficiency. The contrary inference of Livezey (2003) likely reflects skull size being confounded with body size and not corrected for as in our analysis, although we note that at an ordinal level, skull size and the ability to fly appear to be linked (Cubo & Arthur, 2001; Gussekloo & Cubo, 2013). Femur length also does not significantly correlate with flight while femur width does, which expresses the link between femur cross‐sectional area and body mass (Trewick, 1996).

We found three putatively flightless rails had morphological traits that appear closer to the flying rails (the three black dots that fell within the 95% confidence ellipse of the flying rails in Figure 2). There is limited information on the ecology of *Aramidopsis plateni, Megacrex inepta,* and *Gallirallus insignis* (Figure 2) and *Gallirallus insignis* has been described as both almost flightless (del Hoyo et al., 2015) and not flightless (Gilliard, 1967). Given that uncertainty, the lack of information and the principal component analysis could indicate that they have been assigned to the wrong group. They might also be considered as part of an intermediate group including the “almost flightless.” Such a group would also include *Eulabeornis castaneoventris* (the only “flying” species that falls outside the confidence ellipse and closer to the flightless group in Figure 2) and is described as a weak flyer (Taylor, 1998). We found that dividing the rail data into three categories (flying, flightless and almost flightless), or removing *Aramidopsis plateni, Megacrex inepta,* and *Gallirallus insignis* did not significantly change the result (data not shown).

As in some other bird families (Cubo & Arthur, 2001), flighted rails develop their forelimbs more than their hindlimbs as they always exhibit a larger ratio when traits associated with flight (wing length, sternum depth, and length) are divided by traits associated with walking (pelvis width and femur length; Figure 3). This makes sense as we can easily imagine that selective pressures on flightless rails involved the development of powerful hindlimbs to move on the ground rather than the preservation of wings and pectoral muscles of which they have no use (or at least not as much use as their flighted ancestors).

A clear difference is observed between groups in the sternum depth/sternum length ratio (Figure 3). Indeed, flighted rails have a deeper and longer sternum allowing the insertion of powerful pectoral muscles involved in flight. The flightless rails possess a shallower sternum relative to its length. This is not surprising; indeed, sternum reduction is observed in many flightless birds as this bone is associated with flight muscles (Lambertz & Perry, 2015). The interesting part about this ratio difference is that two measurements of the same bone can give an indication regarding the flying ability of a species (Bickley & Logan, 2014). This might be useful in a context of paleontological research where it is not possible to directly assess pectoral muscle mass and has been used in the past to investigate the ability to fly of ancient species based on fossils (Howard, 1964; Trewick, 1997b).

The transition to flightlessness in rails and other bird families appears to show similar trends that indicate a convergent evolution on a much broader phylogenetic scale. Examination of shape differences in volant island bird populations on islands suggests a tendency to change shape in a way that converges subtly on the flightless form (Wright & Steadman, 2012). They highlighted an energy allocation from the forelimbs to the hindlimbs in most of the islands birds (Wright et al., 2016) associated with smaller flight muscles (Wright & Steadman, 2012). In developmental terms, this might be achieved via a neotenic condition mostly manifested as a reduction of the pectoral apparatus and the wings (Olson, 1973). Other traits affected by the loss of flight probably reflect ecological release, such as the tendency for flightless birds to exhibit larger pelves and skulls (Cubo & Arthur, 2001; Gussekloo & Cubo, 2013). Penguins and many flightless ducks do not, however, strictly follow the same morphology trends as their pectoral muscles are not significantly reduced compared with flying birds because of adaptation for aquatic “flying” (McNab, 1994). The basal rate of metabolism is associated with the activity of organisms, and as a consequence, it has been observed that some flightless birds exhibit a lower rate than related flying species. This can be explained by the relative energy costs of aerial and terrestrial locomotion, and it has been shown that the basal metabolic rate increases with the importance of muscles involved in flight (pectoral mass; McNab, 2002).

### 4.2 Phylogeny and evolution

Flightlessness has evolved multiple times in rails (Figures 4 and 5) and as most of the flightless species are endemic to islands, it follows that they all had flighted ancestors who dispersed to these islands (Garcia‐R et al., 2014a, 2014b; Kirchman, 2009). Therefore, the loss of flight probably occurred (at least) as many times as there are flightless species in the phylogenetic tree (Figure 4). This biased proportion (especially from the *Rallus* clade) might be the result of the sampling (we may have more data on flightless birds than on flighted ones) or the consequence of the extinction of most of the flighted ancestors, but it is possible that a few flying ancestors colonized many different islands resulting in a phylogenetic cluster of several flightless island endemic species and only a few flighted ones (Diamond, 1991). It is also possible that flighted lineages do not speciate so readily as the flightless one because they are not as geographically subdivided (Trewick, 1997; Garcia‐R, Gonzalez‐Orozco, & Trewick, 2019).

The large number of flightless rails within the *Rallus* clade might potentially introduce a bias in the morphological analysis. Indeed, the phenotypic trends observed in flightless species could reflect the overall body shape of the *Rallus* clade rather than convergent evolution within flightless rails but the phylogenetic signal test showed this is not the case.

The phylomorphospace (Figure 5) shows the flying–flightless pairs of closely related species can be morphologically very distant. This phenomenon emphasizes that morphology of rails (or at least the morphological characters selected in this analysis) is more affected by the ability to fly than by the phylogeny. This is confirmed by the phylogenetic signal analysis (Table 3). Indeed, none of the morphological trait involved in this study show a significant signal (Blomberg's *K* was always <1). This result is in concordance with Olson (1973) who described the flightless condition as a rapid evolution that involves little genetic modification, and is without major phylogenetic significance.

Flight involves strict physical constraints in term of body size, shape, and weight (Hone, Dyke, Haden, & Benton, 2008; Vizcaíno & Fariña, 1999), which implies that most flighted birds in this analysis have similar shape (though size may differ). Flightless rails on the other hand show wider overall (among species) variation (Figure 2) apparently linked to the fact that most of the flightless species live on islands. Flightless populations on islands have limited exchange (gene flow) so their evolution can be intensely constrained by local selective pressure leading to rapid morphogenesis in response to the environmental conditions (Garcia‐R, 2019). For example, closely related species of *Gallirallus* on the Chatham Islands share reduction in sternum size but show very different responses in body size and relative beak length (Trewick, 1997b). The variation within the flightless group also indicates that the loss of flight results in changes that are not in a single direction. Freed from the constraint of flight, a number of different viable ecological opportunities for trait evolution may emerge leading to the impression of relaxed or nonconstraining evolution. Without the physiological demands of flying, a population can evolve as a function of the ecological opportunities available to them resulting in a wider range of phenotypic outcomes among species (Trewick, 1997b). At the species level, directional evolution is still involved, but when all flightless species are considered, the range of ecological outcomes results in a wide variance of phenotypes and thus appears overall as relaxed selection. In other words, during the transition to flightlessness morphological traits can diverge in many different ways as there are more viable options as a result of less strict morphological constraints.

In conclusion, this study confirms the convergent evolution of multiple morphological traits in flightless rails. Flightless rails exhibit short wings and small sterna as well as wide pelves and femora whereas flying rails have long wings, deep sterna, and smaller femora and pelves. In the rails, we found no evidence that cranial traits are correlated with the ability to fly (c.f. Livezey, 2003), and this likely reflects the correlation between cranium size and overall size of the birds which we corrected for. Using independent molecular phylogenetic data, we show that traits of flightless rails are not subject to phylogenetic constraint but rather reflect a morphological convergent adaptation to the loss of flight.

## CONFLICTS OF INTEREST

None declared.

## AUTHOR CONTRIBUTION

**Julien Gaspar:** Conceptualization (lead); Data curation (lead); Formal analysis (lead); Investigation (lead); Methodology (equal); Software (equal); Writing‐original draft (lead); Writing‐review & editing (equal). **Gillian C. Gibb:** Conceptualization (supporting); Data curation (supporting); Formal analysis (supporting); Funding acquisition (lead); Methodology (supporting); Software (supporting); Supervision (lead); Writing‐original draft (supporting); Writing‐review & editing (equal). **Steven A. Trewick:** Conceptualization (supporting); Data curation (supporting); Formal analysis (supporting); Methodology (supporting); Supervision (lead); Validation (supporting); Writing‐original draft (supporting); Writing‐review & editing (equal).

## Data Availability

Supplementary material can be found on dryad https://doi.org/10.5061/dryad.dz08kprsz and in Appendix 1.

## APPENDIX 1

**TABLE A1:** Full morphological dataset, 97 rail species, missing values for: flying = 0; body length = 14, wing length = 0; body mass = 32; cranial length = 41; cranial depth = 41; cranial width = 36; sternum length = 34; sternum depth = 34; pelvis width = 38; femur length = 38; femur width = 38

Scientific name	In Livezey	Flying	Body length	Wing length	Body mass	Cranial length	Cranial depth	Cranial width	Sternum length	Sternum depth	Pelvis width	Femur length	Femur width
*Zapornia akool*	*Amaurornis akool*	1	270	126.95	133.5	na	na	15.2	na	na	na	na	na
*Megacrex inepta*	*Amaurornis ineptus*	0	370	179.7	967	na	na	27.8	na	na	na	na	na
*Amaurornis isabellina*	*Amaurornis isabellinus*	1	375	165.35	na	na	na	20.3	na	na	na	na	na
*Amaurornis moluccana*	*Amaurornis moluccanus*	1	265	136.1	191.5	30.75	17.85	19.6	37.6	11.35	13.75	47.8	7.85
*Amaurornis olivacea*	*Amaurornis olivaceus*	1	310	162.95	276.5	33	19.95	21	38.45	8.85	16.4	55.05	9.45
*Amaurornis phoenicurus*	*Amaurornis phoenicurus*	1	305	162.15	210	31	18.45	19.6	41.1	11.3	14.05	47.85	8.35
*Amaurornis moluccana ruficrissa*	*Amaurornis ruficrissus*	1	na	140.3	163.5	na	na	na	na	na	na	na	na
*Aramides albiventris*	*Aramides albiventris*	1	na	189.3	466	na	na	na	na	na	na	na	na
*Aramides cajaneus*	*Aramides cajanea*	1	365	180.55	417.5	39.35	23.5	23.25	58	13.05	17.6	62.25	11.45
*Aramides ypecaha*	*Aramides ypecaha*	1	430	223.75	692.5	42.65	26.6	25.95	76.35	17.65	22.75	77.35	17.1
*Aramidopsis plateni*	*Aramidopsis plateni*	0	300	147.4	na	na	na	na	na	na	na	na	na
*Atlantisia rogersi*	*Atlantisia rogersi*	0	142	54.85	38.75	na	na	na	7.7	2.4	9.6	24.3	4.1
*Gallirallus modestus*	*Cabalus modestus*	0	195	83.2	na	25.05	15.2	14.3	11.25	2.65	9.6	30.05	5.05
*Mentocrex kioliodes*	*Canirallus kioloides*	1	280	132.85	250.5	31.5	19.1	19.7	43.5	10.7	17.9	44.3	8.2
*Canirallus oculeus*	*Canirallus oculeus*	1	300	174.15	278	33.5	21.4	21.1	48.3	12.3	18.7	53	9.7
*Coturnicops noveboracensis*	*Coturnicops noveboracensis*	1	175	85.65	55.5	20.45	12.5	12.95	23.8	9.35	9.5	28.7	4.4
*Mustelirallus albicollis*	*Crex albicollis*	1	225	108.65	104	28.15	16.65	17.1	33.25	10.2	11.5	42	6.7
*Crex crex*	*Crex crex*	1	285	137.5	152	28.05	16.4	16.4	43.3	14.1	12.15	45.6	7
*Cyanolimnas cerverai*	*Cyanolimnas cerverai*	0	290	106	na	na	na	na	25.4	4.2	na	na	na
*Dryolimnas cuvieri abbotti*	*Dryolimnas abbotti*	0	na	135.4	na	32.6	18.8	18.3	41.4	11.1	na	na	na
*Dryolimnas cuvieri aldabranus*	*Dryolimnas aldabranus*	0	na	116.7	182.5	30.6	18.1	17.6	30.55	7.85	13.85	43.6	7.8
*Dryolimnas cuvieri*	*Dryolimnas cuvieri*	0	315	150.95	241	33.2	19.9	19.2	44.75	12.4	15.6	51.3	8.9
*Eulabeornis castaneoventris*	*Eulabeornis castaneoventris*	1	500	210.5	687	na	na	na	50.5	12.3	23.4	72.1	13.9
*Fulica alai*	*Fulica alai*	1	390	179.6	495	32.3	20.4	20.2	52.7	15.25	15.25	52.7	9.9
*Fulica americana*	*Fulica americana*	1	385	187.2	610.5	32.8	20.35	19.75	53.8	16	14.75	52.35	10
*Fulica armillata*	*Fulica armillata*	1	470	195.5	912.5	37.3	23.2	22.4	64.6	16.7	17.8	62.7	13.1
*Fulica atra*	*Fulica atra*	1	375	203.1	831.5	35.05	21.7	21	59.1	16.65	15.5	54.45	10.9
*Fulica cornuta*	*Fulica cornuta*	1	495	284.35	1,978.5	42.6	24.4	25	84.8	22.5	26	78.9	16.2
*Fulica gigantea*	*Fulica gigantea*	1	535	266.1	2,355	42.35	25.55	26.2	82.35	20.05	25.3	83.15	18.05
*Gallicrex cinerea*	*Gallicrex cinerea*	1	415	190.2	426	32.3	19.55	19.6	54	14.55	14.7	55.65	9.4
*Gallinula angulata*	*Gallinula angulata*	1	225	136.4	132	na	na	na	na	na	na	na	na
*Gallinula galeata cachinnans*	*Gallinula cachinnans*	1	355	171.2	366.5	31.6	19.45	18.6	48.6	14.5	16.4	52.7	9.4
*Gallinula chloropus*	*Gallinula chloropus*	1	340	167.15	302.5	31.25	19.05	18.1	45.55	14.15	15.65	49.2	8.95
*Gallinula nesiotis comeri*	*Gallinula comeri*	0	250	144.1	513	33.7	22.2	20.5	38.6	8.5	20.7	56.9	11
*Gallinula galeata*	*Gallinula galeata*	1	na	213.75	448.5	na	na	na	na	na	na	na	na
*Gallinula nesiotis*	*Gallinula nesiotis*	0	250	143.5	513	na	na	na	34.6	8.3	na	na	na
*Gallinula chloropus pyrrhorrhoa*	*Gallinula pyrrhorrhoa*	1	na	159.35	na	na	na	na	na	na	na	na	na
*Gallinula galeata sandvicensis*	*Gallinula sandvicensis*	1	na	175.2	na	31	19.2	18.5	42.1	12.8	16.6	51.1	9.5
*Gallinula tenebrosa*	*Gallinula tenebrosa*	1	375	187.85	531.5	34.35	21.55	19.95	59.7	16.35	18.95	61.7	11.75
*Gallirallus australis*	*Gallirallus australis*	0	550	176.65	890	43.1	24.75	24.8	31.95	6.7	21.35	73.75	14.25
*Gallirallus dieffenbachii*	*Gallirallus dieffenbachii*	0	320	121	na	na	na	na	29.05	8.1	17.1	54.6	10.2
*Gallirallus owstoni*	*Gallirallus owstoni*	0	280	119.85	na	33.1	18.8	18.35	31.05	8.25	15.5	52.25	8.85
*Lewinia pectoralis*	*Gallirallus pectoralis‐group*	1	225	97.3	83.5	25.7	15.6	15.1	29.5	9.7	10.8	36.7	5.7
*Gallirallus philippensis*	*Gallirallus philippensis*	1	290	133.9	182	30.85	18.1	17.75	41.4	12.55	13.8	46.95	7.9
*Na*	*Gallirallus sharpei*	1	na	70	na	na	na	na	na	na	na	na	na
*Gallirallus striatus*	*Gallirallus striatus*	1	275	115.4	112.5	28.3	16.4	16.15	38.9	12.1	11.85	43.4	6.95
*Gallirallus wakensis*	*Gallirallus wakensis*	0	235	90.05	na	25.55	15.55	16.9	21.3	5.35	13.2	36	6.65
*Gymnocrex plumbeiventris*	*Gymnocrex plumbeiventris*	1	315	189.25	292	na	25.6	22.7	na	na	na	na	na
*Gymnocrex rosenbergii*	*Gymnocrex rosenbergii*	1	300	194.35	na	na	Na	na	na	na	na	na	na
*Gallirallus torquatus celebensis*	*Habropteryx celebensis*	1	na	148.8	na	na	Na	na	na	na	na	na	na
*Gallirallus insignis*	*Habropteryx insignis*	0	330	144.1	na	na	Na	22.5	na	na	na	na	na
*Gallirallus okinawae*	*Habropteryx okinawae*	0	320	142.5	433	38.5	22	20.4	34.1	8.5	18.7	61.2	11.7
*Gallirallus torquatus sulcirostris*	*Habropteryx sulcirostris*	1	na	149.1	na	na	Na	na	na	na	na	na	na
*Gallirallus torquatus*	*Habropteryx torquatus*	1	340	147.2	245.5	32.65	19.2	19.55	39	11.05	15.85	51.95	9.3
*Habroptila wallacii*	*Habroptila wallacii*	0	350	167.45	na	45.75	25.9	27.9	47.8	6.2	23.15	79.5	14.3
*Laterallus leucopyrrhus*	*Laterallus leucopyrrhus*	1	150	83.3	44.5	22.4	14	14.9	21.3	6.6	9.25	30.5	4.8
*Laterallus spilonota*	*Laterallus spilonotus*	1	155	67.25	40	na	Na	na	na	na	na	na	na
*Nesoclopeus poecilopterus*	*Nesoclopeus poecilopterus*	0	330	160.95	na	na	Na	na	44	8.5	na	na	na
*Pardirallus sanguinolentus*	*Ortygonax sanguinolentus*	1	340	133.6	137	na	Na	na	40.1	12.3	14.3	48	7.5
*Pardirallus nigricans*	*Ortygonax nigricans*	1	280	130.3	179	na	Na	na	na	na	na	na	na
*Gallinula pacifica*	*Pareudiastes pacificus*	0	230	123.3	na	na	Na	na	24	7	na	na	na
*Gallinula silvestris*	*Pareudiastes silvestris*	0	265	149	450	na	Na	na	na	na	na	na	na
*Porphyrio albus*	*Porphyrio albus*	1	na	229	na	na	Na	na	na	na	na	na	na
*Porphyrio hochstetteri*	*Porphyrio hochstetteri*	0	630	235.45	2,718	50.5	31.45	32.6	50.2	7.35	37.1	100.75	21.25
*Porphyrio indicus indicus*	*Porphyrio indicus*	1	na	225.05	na	na	Na	na	na	na	na	na	na
*Porphyrio madagascariensis*	*Porphyrio madagascariensis*	1	na	245.65	598.5	38.75	25.15	24.1	54.5	15.5	22.1	75.15	12.5
*Porphyrio porphyrio*	*Porphyrio porphyrio*	1	440	262.35	984.5	40.2	26.75	24.45	61.2	16.65	24.65	76.2	13.35
*Porphyrio indicus viridis*	*Porphyrio viridis*	1	na	242.85	na	na	Na	na	na	na	na	na	na
*Porphyrio alleni*	*Porphyrula alleni*	1	240	151.85	139.5	28.4	17.95	18.15	33.25	11.05	14.1	46.65	7.4
*Porphyrio flavirostris*	*Porphyrula flavirostris*	1	245	134	93	25.7	15.5	15.75	28.7	na	13.4	38	6.1
*Porphyrio martinica*	*Porphyrula martinica*	1	315	179.25	226.5	31.35	18.85	19.55	38.9	12.5	15.4	73.45	8.4
*Zapornia atra*	*Porzana atra*	0	180	83.05	77	23.65	14.55	15.35	15.35	5.75	11.35	34	5.55
*Zapornia bicolor*	*Porzana bicolor*	1	210	113.5	na	na	Na	na	na	na	na	na	na
*Porzana carolina*	*Porzana carolina*	1	220	106.2	81	23	13.8	14.1	26.6	10.15	10.6	33.5	5.25
*Zapornia flavirostra*	*Porzana flavirostra*	1	210	102.25	83.5	24.9	15.5	16.15	27.1	8.1	10.65	36.8	5.9
*Zapornia monasa*	*Porzana monasa*	0	180	78	na	na	Na	na	na	na	na	na	na
*Zapornia olivieri*	*Porzana olivieri*	1	190	103.75	na	na	Na	na	na	na	na	na	na
*Zapornia palmeri*	*Porzana palmeri*	0	150	58.45	na	20.25	12.8	12.95	11	3.85	9.75	24.9	4.2
*Zapornia pusilla*	*Porzana pusilla*	1	180	88.3	37.5	20.65	12.6	12.6	22.15	8.6	8.75	27.95	4.15
*Zapornia sandwichensis*	*Porzana sandwichensis*	0	145	67.6	na	na	Na	na	na	na	na	na	na
*Zapornia tabuensis*	*Pozana tabuensis*	1	165	79	42	22.4	13.65	14.05	18.9	6.7	9.3	29.4	4.55
*Rallus aquaticus*	*Rallus aquaticus*	1	255	119.1	164.5	27.6	16.45	15.8	35	11.4	11.25	40.95	6.65
*Rallus elegans*	*Rallus elegans‐group*	1	430	163.25	352.5	34.7	19.3	18.6	52.85	16	14.45	58	9.7
*Rallus limicola*	*Rallus limicola*	1	225	101.95	83	25	15.1	14.5	30.15	10.7	10.3	36.35	5.7
*Rallus longirostris*	*Rallus longirostris*	1	330	140.15	295	33.15	18.2	17.8	46.9	14.95	13.6	53.95	8.85
*Rougetius rougetii*	*Rougetius rougetii*	1	300	131.85	195	32.5	Na	18.3	na	na	na	na	na
*Tribonyx mortierii*	*Tribonyx mortierii*	0	465	178.85	1,292.5	42.75	24.75	23.9	52.8	10	25.6	82.45	16.35
*Tribonyx ventralis*	*Tribonyx ventralis*	1	340	208.15	401	34.3	20.45	20.45	52.45	15.6	19.55	55.05	10.95
*Gallirallus lafresnayanus*	*Tricholimnas lafresnayanus*	0	465	181.35	na	na	Na	na	na	7	23.2	68.2	14.1
*Gallirallus sylvestris*	*Tricholimnas sylvestris*	0	360	137.95	na	36.6	21.7	21.25	32.25	8.3	18.45	59.35	11.4

Note

All units in millimetres except body mass (grams), values in black were retrieved from Livezey (2003), and values in blue were retrieved from Handbook of the Birds of the World Alive Online (del Hoyo et al., 2015).

**TABLE A2:** Subset of the dataset present in Appendix 1 including the 52 rails species with phylogenetic information (Garcia‐R et al., 2014a, 2014b) and the 11 morphological traits with the fewest missing values

Species	Taxa	Flying	Body length	Wing length	Body mass	Cranial length	Cranial depth	Cranial width	Sternum length	Sternum depth	Pelvis width	Femur length	Femur width
*Aramides cajaneus*	*Aramide*	1	365	180.55	417.5	39.35	23.5	23.25	58	13.05	17.6	62.25	11.45
*Aramides ypecaha*	*Aramide*	1	430	223.75	692.5	42.65	26.6	25.95	76.35	17.65	22.75	77.35	17.1
*Mustelirallus albicollis*	*Aramide*	1	225	108.65	104	28.15	16.65	17.1	33.25	10.2	11.5	42	6.7
*Pardirallus sanguinolentus*	*Aramide*	1	340	133.6	137	na	na	na	40.1	12.3	14.3	48	7.5
*Pardirallus nigricans*	*Aramide*	1	280	130.3	179	na	na	na	na	na	na	na	na
*Fulica americana*	*Fulica*	1	385	187.2	610.5	32.8	20.35	19.75	53.8	16	14.75	52.35	10
*Fulica armillata*	*Fulica*	1	470	195.5	912.5	37.3	23.2	22.4	64.6	16.7	17.8	62.7	13.1
*Fulica atra*	*Fulica*	1	375	203.1	831.5	35.05	21.7	21	59.1	16.65	15.5	54.45	10.9
*Fulica cornuta*	*Fulica*	1	495	284.35	1,978	42.6	24.4	25	84.8	22.5	26	78.9	16.2
*Fulica gigantea*	*Fulica*	1	535	266.1	2,355	42.35	25.55	26.2	82.35	20.05	25.3	83.15	18.05
*Gallinula angulata*	*Fulica*	1	225	136.4	132	na	na	na	na	na	na	na	na
*Gallinula galeata*	*Fulica*	1	355	171.2	366.5	31.6	19.45	18.6	48.6	14.5	16.4	52.7	9.4
*Gallinula chloropus*	*Fulica*	1	340	167.15	302.5	31.25	19.05	18.1	45.55	14.15	15.65	49.2	8.95
*Gallinula tenebrosa*	*Fulica*	1	375	187.85	531.5	34.35	21.55	19.95	59.7	16.35	18.95	61.7	11.75
*Porzana carolina*	*Fulica*	1	220	106.2	81	23	13.8	14.1	26.6	10.15	10.6	33.5	5.25
*Tribonyx mortierii*	*Fulica*	0	465	178.85	1,292	42.75	24.75	23.9	52.8	10	25.6	82.45	16.35
*Tribonyx ventralis*	*Fulica*	1	340	208.15	401	34.3	20.45	20.45	52.45	15.6	19.55	55.05	10.95
*Amaurornis moluccana*	*Gallicrex*	1	265	136.1	191.5	30.75	17.85	19.6	37.6	11.35	13.75	47.8	7.85
*Amaurornis phoenicurus*	*Gallicrex*	1	305	162.15	210	31	18.45	19.6	41.1	11.3	14.05	47.85	8.35
*Gallicrex cinerea*	*Gallicrex*	1	415	190.2	426	32.3	19.55	19.6	54	14.55	14.7	55.65	9.4
*Megacrex inepta*	*Gallicrex*	0	370	179.7	967	na	na	27.8	na	na	na	na	na
*Coturnicops noveboracensis*	*Laterallus*	1	175	85.65	55.5	20.45	12.5	12.95	23.8	9.35	9.5	28.7	4.4
*Porphyrio hochstetteri*	*Porphyrio*	0	630	235.45	2,718	50.5	31.45	32.6	50.2	7.35	37.1	100.7	21.25
*Porphyrio porphyrio*	*Porphyrio*	1	440	262.35	984.5	40.2	26.75	24.45	61.2	16.65	24.65	76.2	13.35
*Porphyrio alleni*	*Porphyrio*	1	240	151.85	139.5	28.4	17.95	18.15	33.25	11.05	14.1	46.65	7.4
*Porphyrio martinica*	*Porphyrio*	1	315	179.25	226.5	31.35	18.85	19.55	38.9	12.5	15.4	73.45	8.4
*Zapornia akool*	*Porzana*	1	270	126.95	133.5	na	na	15.2	na	na	na	na	na
*Zapornia flavirostra*	*Porzana*	1	210	102.25	83.5	24.9	15.5	16.15	27.1	8.1	10.65	36.8	5.9
*Zapornia pusilla*	*Porzana*	1	180	88.3	37.5	20.65	12.6	12.6	22.15	8.6	8.75	27.95	4.15
*Zapornia tabuensis*	*Porzana*	1	165	79	42	22.4	13.65	14.05	18.9	6.7	9.3	29.4	4.55
*Aramidopsis plateni*	*Rallus*	0	300	151.2	150	na	na	na	na	na	na	na	na
*Crex crex*	*Rallus*	1	285	137.5	152	28.05	16.4	16.4	43.3	14.1	12.15	45.6	7
*Dryolimnas cuvieri*	*Rallus*	1	315	150.95	241	33.2	19.9	19.2	44.75	12.4	15.6	51.3	8.9
*Eulabeornis castaneoventris*	*Rallus*	1	500	210.5	687	na	na	na	50.5	12.3	23.4	72.1	13.9
*Gallirallus modestus*	*Rallus*	0	195	83.2	60	25.05	15.2	14.3	11.25	2.65	9.6	30.05	5.05
*Gallirallus australis*	*Rallus*	0	550	176.65	890	43.1	24.75	24.8	31.95	6.7	21.35	73.75	14.25
*Gallirallus dieffenbachii*	*Rallus*	0	320	121	340	na	na	na	29.05	8.1	17.1	54.6	10.2
*Gallirallus owstoni*	*Rallus*	0	280	119.85	226	33.1	18.8	18.35	31.05	8.25	15.5	52.25	8.85
*Gallirallus philippensis*	*Rallus*	1	290	133.9	182	30.85	18.1	17.75	41.4	12.55	13.8	46.95	7.9
*Gallirallus wakensis*	*Rallus*	0	235	90.05	105	25.55	15.55	16.9	21.3	5.35	13.2	36	6.65
*Gallirallus insignis*	*Rallus*	0	330	144.1	na	na	na	22.5	na	na	na	na	na
*Gallirallus okinawae*	*Rallus*	0	320	142.5	433	38.5	22	20.4	34.1	8.5	18.7	61.2	11.7
*Gallirallus torquatus*	*Rallus*	1	340	147.2	245.5	32.65	19.2	19.55	39	11.05	15.85	51.95	9.3
*Gallirallus lafresnayanus*	*Rallus*	0	465	181.35	na	na	na	na	na	7	23.2	68.2	14.1
*Gallirallus sylvestris*	*Rallus*	0	360	137.95	470	36.6	21.7	21.25	32.25	8.3	18.45	59.35	11.4
*Gallirallus striatus*	*Rallus*	1	275	115.4	112.5	28.3	16.4	16.15	38.9	12.1	11.85	43.4	6.95
*Habroptila wallacii*	*Rallus*	0	350	167.45	na	45.75	25.9	27.9	47.8	6.2	23.15	79.5	14.3
*Lewinia pectoralis*	*Rallus*	1	225	97.3	83.5	25.7	15.6	15.1	29.5	9.7	10.8	36.7	5.7
*Rallus elegans*	*Rallus*	1	430	163.25	352.5	34.7	19.3	18.6	52.85	16	14.45	58	9.7
*Rallus limicola*	*Rallus*	1	225	101.95	83	25	15.1	14.5	30.15	10.7	10.3	36.35	5.7
*Rallus longirostris*	*Rallus*	1	330	140.15	295	33.15	18.2	17.8	46.9	14.95	13.6	53.95	8.85
*Rallus aquaticus*	*Rallus*	1	255	119.1	115.7	27.6	16.45	15.8	35	11.4	11.25	40.95	6.65

Note

All units in millimeters except body mass (grams).

**TABLE A3:** NCBI access numbers used to investigate the maximum likelihood phylogeny

Species name	16S	COI	FGB‐7	RAG‐1	cyt‐b
*Amaurolimnas concolor*		JQ173980.1			
*Amaurornis akool*		FJ661094.1			JQ342141.1
*Amaurornis flavirostra*	KC613979.1	KC614036.1	KC613861.1	KC613913.1	KC614062.1
*Amaurornis moluccana*	KC613981.1	KC614038.1		KC613915.1	KC614064.1
*Amaurornis phoenicurus*	KC613982.1	JQ342118.1	KC613863.1	KC613916.1	KC614065.1
*Anurolimnas fasciatus*	KC614006.1	KC614046.1	KC613884.1	KC613942.1	KC614090.1
*Anurolimnas viridis*	KC614010.1	JQ174052.1	KC613888.1	KC613947.1	KC614094.1
*Aramides axillaris*	KC613978.1	JN801494.1	KC613860.1	KC613912.1	KC614061.1
*Aramides cajanea*	KC613983.1	JN801496.1	KC613864.1	KC613917.1	KC614066.1
*Aramides mangle*	KC613980.1	KC614037.1	KC613862.1	KC613914.1	KC614063.1
*Aramides ypecaha*	KC613984.1	FJ027148.1	DQ881942.1	AY756084.1	KC614067.1
*Aramidopsis plateni*					JQ347988.1
*Aramus guarauna*	DQ485854.1	FJ027151.1	AY695250.1	DQ881798.1	DQ485899.1
*Canirallus beankaensis*					HQ403671.1
*Canirallus kioloides kioloides*					HQ403670.1
*Coturnicops exquisitus*		NC_012143.1			
*Coturnicops noveboracensis*	KC613985.1	DQ433553.1	AY695239.1	KC613918.1	KC614068.1
*Crex crex*	KC613986.1	GU571355.1	KC613865.1	KC613919.1	KC614069.1
*Diaphorapteryx hawkinsi*					KC614124.1
*Dryolimnas cuvieri*	KC613987.1	KC614039.1	KC613866.1	KC613920.1	KC614070.1
*Eulabeornis castaneoventris*	KC613988.1	KC614058.1	KC613867.1	KC613921.1	KC614071.1
*Fulica alai*	KC613989.1	JF498857.1	KC613868.1	KC613922.1	KC614072.1
*Fulica americana*		DQ434598.1	AY695244.1	KC613923.1	DQ485910.1
*Fulica ardesiaca*	KC613990.1	FJ027587.1	KC613869.1	KC613924.1	KC614073.1
*Fulica armillata*	KC613995.1	FJ027588.1	KC613874.1	KC613929.1	KC614078.1
*Fulica atra*	KC613991.1	GU571406.1	KC613870.1	KC613925.1	KC614074.1
*Fulica cornuta*		FJ027592.1			KC614075.1
*Fulica cristata*	KC613992.1	KC614040.1	KC613871.1	KC613926.1	
*Fulica gigantea*		FJ027593.1			
*Fulica leucoptera*	KC613993.1	KC614060.1	KC613872.1	KC613927.1	KC614076.1
*Fulica rufifrons*	KC613994.1	FJ027594.1	KC613873.1	KC613928.1	KC614077.1
*Gallicrex cinerea*	KC613997.1	JQ342129.1	KC613877.1	KC613932.1	KC614080.1
*Gallinula angulata*	KC613996.1	KC614041.1	KC613875.1	KC613930.1	KC614079.1
*Gallinula chloropus*		FJ027609.1	AY695245.1	KC613931.1	DQ485911.1
*Gallinula galeata sandvicensis*		JF498859.1			
*Gallinula melanops*	KC613998.1	FJ027612.1	KC613878.1	KC613933.1	KC614081.1
*Gallinula mortierii*	KC613999.1	KC614042.1		KC613934.1	KC614082.1
*Gallinula tenebrosa*	KC614002.1	JQ174909.1	KC613880.1	KC613938.1	KC614086.1
*Gallinula ventralis*	KC614003.1		KC613881.1	KC613939.1	KC614087.1
*Gallirallus australis*	KC614035.1		KC613911.1	KC613977.1	KC614123.1
*Gallirallus calayanensis*					KC614128.1
*Gallirallus dieffenbachii*					KC614127.1
*Gallirallus insignis*					JQ347978.1
*Gallirallus lafresnayanus*					KC614130.1
*Gallirallus modestus*					KC614125.1
*Gallirallus okinawae*		NC_012140.1			NC012140
*Gallirallus owstoni*	KC614000.1	KC614043.1		KC613935.1	
*Gallirallus philippensis*			AY695241.1	KC613936.1	DQ485907.1
*Gallirallus rovianae*					JQ348011.1
*Gallirallus striatus*	KC614001.1	JQ342122.1	KC613879.1	KC613937.1	KC614085.1
*Gallirallus sylvestris*	KC614034.1	KC614057.1	KC613910.1	KC613976.1	KC614122.1
*Gallirallus torquatus torquatus*					JQ347980.1
*Gallirallus wakensis*					JQ348014.1
*Habroptila wallacii*					JQ347984.1
*Heliornis fulica*	DQ485857.1	JQ175018.1	AY695246.1		DQ485902.1
*Himantornis haematopus*					KC614126.1
*Laterallus albigularis*		JQ175222.1	AY082411.1	DQ881813.1	
*Laterallus exilis*	KC614004.1	JQ175223.1	KC613883.1	KC613941.1	KC614089.1
*Laterallus jamaicensis*	KC614009.1	DQ432997.1	KC613885.1	KC613943.1	KC614091.1
*Laterallus melanophaius*	DQ485859.1		AY695238.1	KC613944.1	DQ485906.1
*Lewinia mirifica*	KC614005.1	KC614045.1	KC613882.1	KC613940.1	KC614088.1
*Lewinia muelleri*	KC614007.1	KC614047.1	KC613886.1	KC613945.1	KC614092.1
*Lewinia pectoralis*	KC614008.1	KC614048.1	KC613887.1	KC613946.1	KC614093.1
*Megacrex inepta*					JQ347987.1
*Neocrex erythrops*	KC614011.1	KC614050.1	KC613889.1	KC613948.1	KC614095.1
*Nesoclopeus woodfordi*	KC614012.1		KC613891.1	KC613949.1	KC614096.1
*Pardirallus maculatus*		JQ175674.1		KC613965.1	KC614114.1
*Pardirallus nigricans*	KC614020.1	KC614054.1	KC613898.1	KC613957.1	KC614104.1
*Pardirallus sanguinolentus*	KC614025.1	JQ175676.1	KC613904.1	KC613963.1	KC614113.1
*Porphyrio alleni*	KC614015.1	KC614052.1	KC613893.1	KC613952.1	KC614100.1
*Porphyrio hochstetteri*	NC_010092.1	NC_010092.1	KC613909.1	KC613974.1	NC010092
*Porphyrio martinica*	KC614019.1	AY666523.1	KC613897.1	KC613956.1	KC614103.1
*Porphyrio porphyrio*	DQ485858.1	JQ175970.1	AY695240.1	KC613975.1	DQ485905.1
*Porzana albicollis*	KC614018.1	JQ175972.2	KC613896.1	KC613955.1	KC614102.1
*Porzana carolina*	DQ485862.1	DQ433143.1	KC613899.1	KC613958.1	DQ485909.1
*Porzana flaviventer*		JQ175973.1			
*Porzana fluminea*	KC614016.1	KC614053.1	KC613894.1	KC613953.1	KC614107.1
*Porzana fusca*	KC614017.1	JQ342114.1	KC613895.1	KC613954.1	KC614101.1
*Porzana parva*	KC614022.1		KC613901.1	KC613960.1	KC614106.1
*Porzana paykullii*	KC614013.1	JQ342128.1	KC613892.1	KC613950.1	KC614097.1
*Porzana porzana*	KC614023.1	GQ482558.1	KC613902.1	KC613961.1	
*Porzana pusilla*	KC614021.1	JQ342132.1	KC613900.1	KC613959.1	KC614105.1
*Porzana tabuensis*	KC614026.1			KC613964.1	
*Porzana spiloptera*		JN801952.1			
*Psophia crepitans*	DQ485855.1	JQ176018.1	AY695248.1		DQ485900.1
*Rallina eurizonoides sepiaria*	NC_012142.1	NC_012142.1			NC012142
*Rallina fasciata*	KC614030.1			KC613969.1	KC614118.1
*Rallina tricolor*	KC614032.1	KC614056.1	KC613907.1	KC613972.1	KC614120.1
*Rallus aquaticus*	KC614027.1	GU097233.1	EF552781.1	KC613966.1	KC614115.1
*Rallus caerulescens*	KC614028.1	KC614055.1	KC613905.1	KC613967.1	KC614116.1
*Rallus elegans*	KC614029.1	AY666315.1	KC613906.1	KC613968.1	KC614117.1
*Rallus limicola*	KC614031.1	GU097263.1	AY695242.1	KC613970.1	KC614119.1
*Rallus longirostris*	DQ485861.1	DQ433164.1	AY695243.1	KC613971.1	DQ485908.1
*Sarothrura rufa*	KC614033.1		KC613908.1	KC613973.1	KC614121.1
*Grus americana*	KP966312.1	DQ433674.1	AY695254		
